# Clinical educators’ perspectives about use of digital tools and Challenges in their Use in clinical teaching and patient care: A qualitative study

**DOI:** 10.12669/pjms.42.7.13997

**Published:** 2026-07

**Authors:** Sabeen Saad, Saad Ahmed, Muhammad Nasir Ayub

**Affiliations:** 1 Sabeen Saad, FCPS, MHPE Shifa School of Health Professions Education, Shifa Tameer-e-Millat University, Islamabad, Pakistan.; 2 Saad Ahmed, FCPS Department of Anesthesia, Shifa Tameer-e-Millat University, Islamabad, Pakistan.; 3 Muhammad Nasir Ayub Khan, FCPS, PHD Department of Anesthesia, Shifa Tameer-e-Millat University, Islamabad, Pakistan.

**Keywords:** Clinical educators, Digital competence, Digital transformation, Health professions education, Professional development, Qualitative study, Technology integration, Teaching and learning

## Abstract

**Objectives:**

To explore how clinical educators integrate digital tools in teaching and patient care and identify challenges and strategies influencing their digital competence.

**Methodology:**

A qualitative exploratory study was conducted from October 2024 to August 2025. Using maximum variation purposive sampling, postgraduate clinical educators (≥2 years’ experience) across various specialties from three tertiary care hospitals of Islamabad, Pakistan were interviewed after informed consent. Data was transcribed and thematic analysis was done.

**Results:**

Three themes emerged: (1) Impact of Digital Tools-confidence and teaching influence; (2) Barriers and Challenges-technological constraints and resistance; (3) Sustainable Practice-emotional readiness and institutional support.

**Conclusion:**

Clinical educators showed varied digital competence. While digital tools offer significant potential to enhance learning and patient care, they also introduce contextual challenges, highlighting the need for institutional support and professional development for sustainable digital integration.

## INTRODUCTION

With the growing digitalization of healthcare systems, clinical educators must enhance their digital skills to teach medical students and integrate technology into clinical and educational practices, effectively.^1^ However, nearly 58% of healthcare workers report having received no formal training in digital health tools, contributing to low proficiency and reluctance to adopt new technologies.^2^,^3^ In a survey of 498 Indian health profession educators, 93% rated their digital competence below the expert level, with only 7% demonstrating advanced proficiency.^4^ While many educators express positive attitudes toward technology in clinical teaching, their perceptions of its educational impact remain mixed.^5^ Some regard digital tools as valuable pedagogical aids, whereas others questioned their long-term relevance and practical benefits. A qualitative study conducted across six European universities (2022) revealed that although faculty adapted to online modalities, many faced technological, pedagogical, and social challenges that hindered effective practical teaching.^6^

Literature underscored the need to further explore contextual enablers and barriers influencing the integration of digital tools in clinical teaching.^7^,^8^ Clinical educators, while operating in complex healthcare environments, face unique barriers such as balancing patient care with teaching, managing time constraints, and coping with limited technological infrastructure.^9^,^10^ These challenges emphasized the importance of moving beyond surface-level assessments of digital competence to understand the lived realities of educators as they navigate digital transformation in their teaching practices. Despite a growing body of research on digital readiness, limited qualitative inquiry is focused specifically on the day-to-day experiences of clinical educators, particularly in resource-constrained contexts.^11^,^12^

To address this gap, the present study adopts an exploratory qualitative approach grounded in the lived experiences of clinical educators. It seeks to understand how clinical educators perceive the use of digital tools in teaching, what challenges and barriers they face, and how these experiences shape digital integration across clinical learning environments? By capturing their perceptions, challenges, and contextual realities, the study aims to provide insights that can guide institutional support and professional development for effective and sustainable digital transformation in clinical education.

## METHODOLOGY

**Fig.1:**
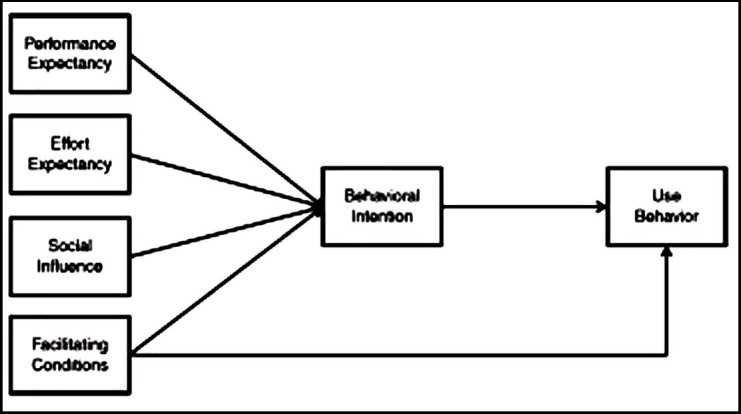
Unified Theory of Acceptance and Use of Technology (UTAUT)

This qualitative exploratory study was conducted from October 2024 to August 2025 from three tertiary care hospitals of Islamabad, Pakistan were interviewed after informed consent. In this study clinical educators’ perceptions and challenges in using digital tools for clinical teaching. The study was conducted in three tertiary care teaching hospitals (two public and one private) using purposive sampling with maximum variation. Twenty postgraduate clinical educators with at least two years of experience in clinical instruction and digital tool use were recruited. Participant selection was conducted iteratively and reviewed within the research team to minimize individual selection bias. This Study was guided by Unified Theory of Acceptance and Use of Technology (UTAUT), explaining factors influencing technology adoption across four domains: performance expectancy (perceived usefulness), effort expectancy (ease of use), social influence, and facilitating conditions. (Fig.1).^13^ Data was collected through semi-structured interviews based on UTAUT constructs interview guide was reviewed and validated by two independent medical educationists to ensure alignment (Supplementary File.1). These Interviews lasted for 30-45 minutes and were conducted in-person or online. They were recorded after taking informed consent, and transcribed verbatim using Otter.ai. Data was analyzed thematically following Braun and Clarke’s six-step framework,^14^ combining deductive (UTAUT-based) and inductive coding. Credibility was ensured through member checking with eight participants who reviewed transcripts for accuracy, with minor clarifications incorporated. An audit trail documenting data, coding, and theme development supported transparency. Peer debriefing with an independent qualitative research expert in health professions education enhanced analytical rigor and minimized bias.^15^ Ethical approval was obtained from Institutional Review Boards (IRB# 493-24, October 16, 2024).

**Supplementary File.1:** Alignment of Interview Guide with UTAUT Constructs.

UTAUT Construct	Description	Sample Interview Questions
Performance Expectancy (PE)	The extent to which clinical educators perceive that digital tools enhance teaching effectiveness, patient care, and learning outcomes.	1. What impact do digital tools have on your teaching effectiveness?
		2. How do digital technologies influence students’ understanding and retention of concepts?
		3. Do digital tools help engage students during clinical sessions? How?
		4. How do you use digital tools in patient care?
		5. In what ways do digital tools improve (or not improve) your effectiveness in teaching or clinical practice?
Effort Expectancy (EE)	The perceived ease or difficulty of learning and using digital tools in teaching and clinical settings.	6. How confident do you feel using digital tools in your teaching or clinical work?
		7. How easy or difficult do you find learning and using digital tools?
		8. What challenges do you face when using digital tools?
		9. How do time constraints or technical issues affect your use of digital technologies?
Social Influence (SI)	The extent to which colleagues, students, and institutional expectations influence the use of digital tools.	10. Have you experienced resistance or support from colleagues or students when using digital tools?
		11. How do peers or supervisors influence your use of digital technologies?
		12. Do institutional expectations affect your decision to use digital tools?
Facilitating Conditions (FC)	The availability of institutional, technical, and training support that enables the use of digital tools.	13. What kind of support (technical, administrative, or training) is needed to integrate digital tools effectively?
		14. Have you received formal training in digital tools? What was beneficial?
		15. How does your institution support digital integration?
		16. What additional resources or changes would improve your use of digital tools?
Behavioral Intention (BI)	The willingness of clinical educators to adopt or expand the use of digital tools in the future.	17. What digital tools would you like to incorporate in the future?
		18. Can you describe an innovative way in which you can integrate digital technologies into your clinical education or healthcare setting?

## RESULTS

Our results demonstrate a continuum of digital engagement, moving from perceived impact and use to experienced barriers, and ultimately to strategies for sustainable integration in clinical teaching (Table-I).

**Table-I:** Themes and subthemes with alignment with theoretical framework.

Themes	Subthemes	UTAUT Link
Perceived impact of digital tools	Varied confidence across digital tools Enhancing teaching effectiveness through digital integration Shaping students’ understanding in digital environments Challenges in patient engagement through digital education tools	**Performance Expectancy.** *(The belief that using technology will improve job performance or make tasks more effective)*
Perceived Barriers and Challenges	Technological complexities Contextual clinical barriers. Resistance to change Learning modifiers	**Effort Expectancy** *(The perceived degree of ease/difficulty associated with using technology)* ***Behavioral intentions/Facilitating Conditions*** *(The degree to which an individual believes that organizational and technical infrastructure exists to support the use of technology.)* ***Social Influence*** *(The extent to which people perceive that important others (peers, supervisors, students) expect them to use the technology).*
Strategies for Sustainable clinical Digital Teaching	*Emotional and Psychological Readiness Support* *Clinical KPIs or Appraisals for Digital Teaching* *Micro competencies* *Digital Pedagogy Anchored in Clinical Scenarios* *Learning by Designing*	

### Theme-1: Perceived impact of digital tools:

Participants reported a broad range of confidence levels in using digital tools. While most felt comfortable with basic technologies like PowerPoint, Zoom, or WhatsApp, they expressed hesitation when using more advanced or interactive platforms. This variability contributed to inconsistent use of digital technologies in both educational and clinical settings.

*“I feel confident using basic tools like PowerPoint or WhatsApp, but anything beyond that, I hesitate because I’m not sure I’ll get it right.”* – *Participant 2* *“My confidence depends on the tool. I’m fine with Zoom or Teams, but when it comes to using apps for interactive learning, I feel lost.”* – *Participant 6*

Several Participants acknowledged that the use of digital tools significantly enhanced their teaching effectiveness by enabling more interactive, engaging, and accessible learning experiences. These tools allowed for better visualization of complex concepts and more timely feedback for students.

*“Students seemed more engaged when I introduced the digital simulations. They asked more questions, stayed longer after class, and told me they finally understood the procedure clearly” participant 14*

Many educators felt that integrating digital technology supported their ability to deliver content in a more structured and student-centered way. Quizzes, multimedia, and visual aids were especially valued

*“Using digital platforms has completely changed how I teach. I can reach students more effectively, explain difficult topics with visual aids, and keep them actively involved throughout the session.” Participant 12*

Similarly, majority Educators who had integrated digital tools into their teaching reported improved student engagement, content clarity, and more interactive learning experiences.

*“Digital tools enhance student engagement, allow quick feedback if we’re using quizzes, and help students learn at their own pace.” – Participant 13*

However, those few with lower digital competence often defaulted to traditional methods due to fear of technical failure or lack of familiarity

*“I stick to the basics because I don’t want to risk trying something digital that might not work in the middle of a session.” – Participant 12*

Majority educators recognized that digital tools could support patient education by simplifying complex medical concepts. However, their practical use remained minimal, often limited to WhatsApp for sharing lab results.

*“Our digital interaction is minimal. Mostly we use WhatsApp for sharing lab reports or addressing patient concerns, but secure channels or dedicated tools are just wishful thinking without institutional support” – Participant 6*

This seamless integration of teaching and patient care presents several contextual and operational challenges that hinder consistent digital adoption

### Theme 2: Perceived barriers and challenges:

Participants described multiple barriers that complicated the adoption of digital tools in their clinical teaching environments. Successful digital adoption was perceived to depend on certain technology related features such as “*ease of technology use***”, “***adequate adaptation time***” and “***user-friendly design”***,** were seen as important facilitators.

*“When the learning process is straightforward and the help is available, I’m much more open to trying new tools.”* *– Participant 16*.

*“If I can get comfortable with a new tool quickly, it’s far more likely I’ll keep using it.” –* Participant 18

*The most significant barrier to digital adoption, reported by majority of the participants, was the lack of workflow and mobility fit of the digital tools, as the fast-paced, unpredictable clinical environment and heavy patient load left little room to integrate digital tools into teaching, despite participants’ preference for tools that align with existing routines*.

*“I need something that works with my schedule and that I can easily access on the go—it makes teaching much more flexible.” – Participant 11*

*“In the middle of a busy clinic, I simply don’t have the time to set up and manage these digital systems”* *– Participant* 14.

Recurrent theme was Socio-cognitive pressures that significantly influence educators’ technology adoption behaviors in clinical practice. Most educators reported cognitive overload (tech fatigue) and dual role conflict, as they balanced clinical care, teaching, and digital documentation.

*“By the time I finish updating everything in the system, my brain is already tired—then I must explain things to students and patients. It’s like running two parallel jobs.” – Participant 5*.

*Managing patients, teaching students, and handling digital tasks together makes it feel like I’m performing three jobs at once” – Participant 12*

Followed by perceptions of digital work as “invisible labor”, contributing to frustration and burnout.

*“All this digital teaching prep and online coordination—no one counts it as part of our workload. It’s like invisible work that just drains you.” – Participant 2*.

*Emotional resistance* to digital adoption was particularly evident among more experienced educators, who were hesitant to shift from established teaching practices. This was driven by comfort with traditional methods, skepticism about the value of new technologies, erosion of clinical skills by use of digital tools and fear of public failure or loss of authority, when using unfamiliar tech

*“I sometimes worry that relying too much on digital simulations might erode the hands-on clinical skills that students really need to develop at the bedside” –* Participant 6.

*“I’ve been teaching the same way for years, and sometimes I question whether these digital tools really add enough to justify the effort to change” – Participant 12*.

*“It’s not just about learning the tools—it’s about feeling confident enough to use them without fear of failure or judgment” – Participant 12*.

Many participants relied strongly on self-directed learning in the absence of formal institutional support for digital literacy. Educators commonly learned through trial and error, using resources on internet. One participant noted,

*“I simply don’t know where to begin, which often leaves me navigating the learning process on my own through trial and self-directed efforts “ – Participant 6*.

Peer and senior faculty influence, along with institutional and policy-driven pressures, emerged as key drivers of digital adoption. Observing respected colleagues using digital tools encouraged uptake, while institutional expectations further pushed educators to integrate technology into their teaching. A participant explained,

*“When I see my seniors using these tools confidently, I feel encouraged to try them myself” – Participant 15*

*“It’s not that I’m unwilling, I just don’t know where to start with some of these tools, and the institution now expects us to be using them.” – Participant 6*

Finally, student expectations and preferences significantly shaped teaching practices, as educators felt increasing pressure to meet the digital demands of tech-savvy learners. As one participant shared,

*“Students today expect digital learning—it pushes us to keep up whether we’re ready or not.” – Participant 16*

Collectively, these barriers contributed to hesitation and selective adoption of digital tools among clinical educators. Despite these difficulties, clinical educators expressed optimism toward future digital transformation and proposed practical strategies to strengthen and sustain digital integration within clinical teaching practices

### Theme 3:

Strategies for Sustainable clinical Digital Teaching: The need for emotional and psychological readiness support was highlighted, emphasizing that confidence and a safe learning environment are essential for adopting digital tools without fear of failure or judgment.

*“It’s not just about learning the tools—it’s about feeling confident enough to use them without fear of failure or judgment.” – Participant 12*

Similarly, the digital skills needed to be formally recognized within clinical performance indicators (KPIs) and faculty evaluations. Despite growing expectations for technology integration, these efforts remain largely “invisible” in appraisal systems.

*“We’re expected to innovate digitally, but when appraisal time comes, none of that work counts. It should be part of how our teaching is assessed” – Participant 13*.

Educators emphasized the need for flexible, bite-sized, and competency-based digital learning modules targeting specific skills such as virtual OSCEs and interactive content design. These micro-competencies were valued for enabling self-paced learning, confidence building, and feasibility within time-constrained clinical environments.

*“Short, focused modules work best for us. I don’t need a full course—I just need to know how to make a virtual case work well for my students” – Participant 20*.

At the same time, participants stressed that digital pedagogy should be *grounded in authentic clinical contexts*, integrating tools like virtual cases and video consultations into real clinical scenarios to enhance relevance, learner engagement, and clinical reasoning.

*“When the digital content mirrors our actual clinical cases, it’s no longer just e-learning—it becomes part of how we teach medicine on the floor” – Participant 15*.

Similarly, educator’s engagement and digital teaching skills would be enhanced through active participation in the design and development of digital learning tools, including interactive cases, quizzes, and applications—customized to their specific instructional contexts, rather than relying solely on pre-existing resources.

*“I know there are multiple tools for almost every task out there, but they all do not fit for purpose. I think they all need customization and that’s what I do not know how to do?” – Participant 6*.

## DISCUSSION

The present study contributes to the growing understanding of digital competency among clinical educators. The findings of this study reveal that while clinical educators recognize the value of digital tools in enhancing teaching effectiveness, student engagement, and patient education, their confidence and adoption remain uneven. Similar patterns have been reported in studies by Mainz et al. (2022), where educators valued digital tools but faced difficulties in integrating them.^16^ Our results are similar with that of another study,^17^ which places the technical level of medical teachers above the pedagogical level. At the same time, it has been shown that the teachers survey scores place them at intermediate levels of digital competencies, which is comparable to moderate confidence levels observed in our study population.^18^ Research on the UK Allied Health Professionals Digital Competency Framework found that over 50% of participants rated their digital skills as poor, particularly in decision support, digital leadership, and EHR use. Similarly, our study identified deficiencies in technologically empowering students, consistent with Cabero et al.^19^ Clinical educators in this study encountered multiple barriers to adopting digital tools, which include previously established barriers such as limited time, insufficient institutional support, and lack of formal training.^16^,^20^

This study identifies several unique “*technology-related barriers*” specific to clinical environments that have not been previously reported. A key finding is that “*ease of technology integration”* strongly influences educators’ willingness to adopt digital tools, which aligns with the UTAUT construct of *effort expectancy* and prior evidence that perceived ease of use is a critical determinant of technology acceptance among healthcare educators. Many highlighted the importance of “*learning accessibility”*, noting that when digital platforms are simple to understand and supported by adequate training, they feel more confident in using them.^19^ Similarly, “user friendly design” and “on the go design” were also given weightage as it helps seamless integration in busy on-the-go clinical lifestyle.^21^ In a systematic review also emphasizes that user-centered clinical software design, workflow compatibility, usability, and integration into real-time clinical practice are critical factors influencing adoption and sustained engagement with digital systems among clinicians and educators.

Similarly, contextual challenges within clinical environments-such as *workflow disruptions* further constrained digital engagement, echoing findings from Svendsen et al. that excessive digital multitasking can impede both teaching quality and clinical performance.^22^ Additionally, Emotional and psychological readiness is crucial for integrating digital tools into clinical teaching. The socio-cognitive barriers specifically in clinical spaces, such as “*dual role conflict*” and cognitive overload (tech fatigue) further hindered adoption; as educators described feeling torn between patient care and teaching duties, often perceiving digital engagement as an added workload, as endorsed by a systematic review as well 23. *Resistance to change* driven by comfort with traditional teaching approaches and concerns about the potential erosion of clinical skills in digitally mediated environments emerged as a significant barrier, which intersect with both performance and effort expectancy, and further shaped attitudes toward technology use.^24^ These findings are consistent with literature emphasizing the psychological and professional identity challenges faced by educators navigating digital transformation.^25^ The notion of “invisible labor” highlights gaps in facilitating conditions, where educators’ digital teaching efforts remain unrecognized, reducing motivation for sustained adoption.^26^

Learning facilitators such as peer modelling, student expectations, and institutional mandates reflect social influence, acting as both enablers and stressors. Saira Soomro et al also argued that educators were more inclined to adopt digital tools when inspired by respected colleagues or motivated by tech-savvy students. However, the absence of formal support systems led many to rely on informal, trial-and-error learning approaches.^20^ Collectively, these barriers underscore that successful digital adoption in clinical education requires not only technical readiness but also on alignment with facilitating conditions.^20^

The lack of formal recognition for digital teaching remains a major demotivator, as such efforts are often excluded from clinical key performance indicators (KPIs) or appraisals, echoing Shon et al. and Soomro et al, who advocate integrating digital pedagogy into educator evaluations to legitimize and sustain innovation.^20^,^24^ To address the practical constraints of clinical educators, educators emphasized the need for short, stackable micro-competency modules, that allow targeted skill development such as designing virtual OSCEs or creating interactive video content. This flexible, modular approach reflects shifts in faculty development toward personalized, time-efficient learning formats.^23^ Moreover, educators advocated for digital pedagogy that is clinically anchored, whereby digital content is directly contextualized within real-life patient care scenarios.^28^ Educators valued learning through designing, seeking to co-create digital tools tailored to clinical settings. This participatory approach, as endorsed by Paskevicius et al., fosters ownership and aligns with constructivist models emphasizing educators as creators, not just consumers of digital content.^28^ Overall, digital transformation in clinical education is shaped by technological, emotional, and institutional factors, requiring supportive environments that recognize digital teaching as integral to professional practice.

### Limitations

The participant pool was limited as study was done in specific institutions, which may affect the transferability of the findings to other clinical or educational contexts. Moreover, as participation was voluntary, there is a potential for response bias, with individuals more engaged or interested in digital teaching being overrepresented. Additionally, the fast-paced evolution of digital tools and institutional strategies means that some findings may lose relevance over time. Gaining insights from other health professionals, patients, and students can provide a more comprehensive and well-rounded understanding of the required competencies.

## CONCLUSION

This study highlights the dual burden faced by clinical educators in balancing teaching and patient care while engaging with digital tools, reflecting varying levels of digital competence. Digital integration emerged not merely as a technical process but as a cultural and emotional shift, shaped by cognitive-emotional, technical constraints, and clinical workflow barriers. Peer modeling, prior exposure, students expectations and institutional mandate acted as enabling factors, underscoring the need for active involvement of clinicians in learning, designing and implementing context-sensitive, job-specific learning experience and faculty development that addresses both technical and psychosocial dimensions of digital adoption.

### Future recommendations:

Faculty development should adopt tiered, flexible training models grounded in real clinical teaching contexts. Institutions should foster psychological safety to support experimentation and sustain digital skill development.
